# Case Report: Diagnosis of cervical carcinoma from pelvic tumor during pregnancy

**DOI:** 10.3389/fmed.2025.1648261

**Published:** 2025-08-12

**Authors:** E. A. Hase, M. C. M. Cruz, M. M. Kondo, M. T. A. Barbosa, J. C. Sadalla, F. Gabrielli, M. D. P. E. Diz, S. A. C. Siqueira, R. P. V. Francisco

**Affiliations:** ^1^Department of Obstetrics and Gynecology, Hospital das Clínicas, Faculty of Medicine, University of São Paulo Medical School, São Paulo, Brazil; ^2^Department of Gynecologic Oncology, Institute of Cancer of São Paulo, São Paulo, Brazil; ^3^Department of Pathological Anatomy, Hospital das Clínicas, Faculty of Medicine, University of São Paulo Medical School, São Paulo, Brazil

**Keywords:** cervical cancer, carcinoma, pregnancy, tumor, neoplasm

## Abstract

Cervical cancer, the fourth most prevalent cancer in women, is the most common malignant tumor of the female reproductive system and one of the most frequently diagnosed cancers during pregnancy. In Brazil, cervical cancer screening is conducted via Pap smear examination of the cervix, with a false-negative rate ranging between 2 and 50%. Colposcopy is recommended when the Pap smear results reveal abnormalities suggestive of malignancy, and biopsy is reserved for patients who are pregnant only when invasive lesions are suspected. We report the case of a pregnant woman who developed a tumor in the inguinal region, which was biopsied and diagnosed as squamous cell carcinoma. An investigation was performed to identify the primary focus of the tumor. Since the Pap smear was normal, two other potential primary sites were investigated, including the colorectal and bladder regions, which were normal. Despite normal cervical cytopathological examination results and due to the high suspicion of a primary cervical focus, additional diagnostic evaluations were performed, which confirmed cervical squamous cell carcinoma as the primary site. We described this case due to the lack of similar reports in the literature of cervical cancer diagnosed in pregnant or non-pregnant patients with normal oncotic Pap smears, initially diagnosed by biopsy of an inguinal tumor, and to highlight the importance of complementing the diagnostic process with colposcopy in patients with a high clinical suspicion of cervical cancer, even in cases of negative Pap smear results.

## Introduction

A cancer diagnosis during pregnancy is defined as one made during pregnancy or within 1 year postpartum. Cervical cancer is the second most frequently diagnosed neoplasm during pregnancy or the postpartum period, being one of the most common malignancies in this context (1). Globally, cervical cancer is the fourth most prevalent cancer among women (2) and the leading malignant tumor of the female reproductive system (3). In 2020, cervical cancer accounted for 604,127 new cases and 341,831 deaths worldwide (2).

In our context, cervical cancer screening is performed via cervical cytology (Pap smear), beginning at 25 years of age (4). The false-negative rate of this test ranges between 2 and 50% (5), influenced by errors in the pre-analytical, analytical, and post-analytical phases. These include inadequate sample collection, misinterpretation, improper slide staining and preparation, or insufficient expertise of the cytologist (6). For pregnant women, the screening recommendations regarding frequency and age range are identical to those for the non-pregnant women, and prenatal care should be considered an opportunity to provide screening services. When Pap smear results reveal abnormalities suggestive of malignancy, colposcopy is indicated, and biopsy is reserved for cases in which an invasive lesion is suspected (4).

The primary symptoms of cervical cancer include genital bleeding, dyspareunia, abdominal pain, pelvic pain, and abnormal vaginal discharge (2). However, in this study, the patient sought medical care due to progressive edema of the right lower limb associated with severe localized pain, which partially improved with over-the-counter analgesics. An ultrasound of the right inguinal region identified a solid, hypoechoic, heterogeneous lesion with irregular contours. A biopsy of the lesion in the right hip and thigh base revealed an invasive, poorly differentiated carcinoma with necrotic areas. Despite a normal Pap smear 1 year before, a new test was performed, which was again negative for malignancy. The literature has no reported cases of cervical cancer diagnosed in either pregnant or non-pregnant patients with normal oncotic Pap smear initially diagnosed via biopsy of an inguinal tumor. Thus, this study aims to highlight the importance of complementing the diagnostic process with colposcopy in patients with a high clinical suspicion of cervical cancer, even in cases of negative Pap smear results.

### Case description

A 30-year-old woman in her fourth pregnancy, with three previous full-term vaginal deliveries, presented to the Obstetrics Clinic of the Hospital das Clínicas of the Faculty of Medicine of the University of São Paulo (HC-FMUSP) at 12 weeks of pregnancy. She reported progressive edema of the right lower limb for 6 months associated with severe localized pain, which partially improved with over-the-counter analgesics. She had previously sought medical care at other facilities to investigate the pain, where she underwent magnetic resonance imaging (MRI) of the right thigh. MRI revealed iliac and inguinal lymph node enlargement of up to 4.3 cm, as well as solid tissue invasion of the right iliac muscles and medial wall of the acetabulum, and diffuse subcutaneous edema in the thigh.

Upon admission, physical examination revealed a mass in the right inguinal region, suggestive of lymph node enlargement, along with significant asymmetric edema of the right lower limb, without associated hyperemia. The patient was hospitalized for pain management and further diagnostic evaluation.

**Figure 1:**
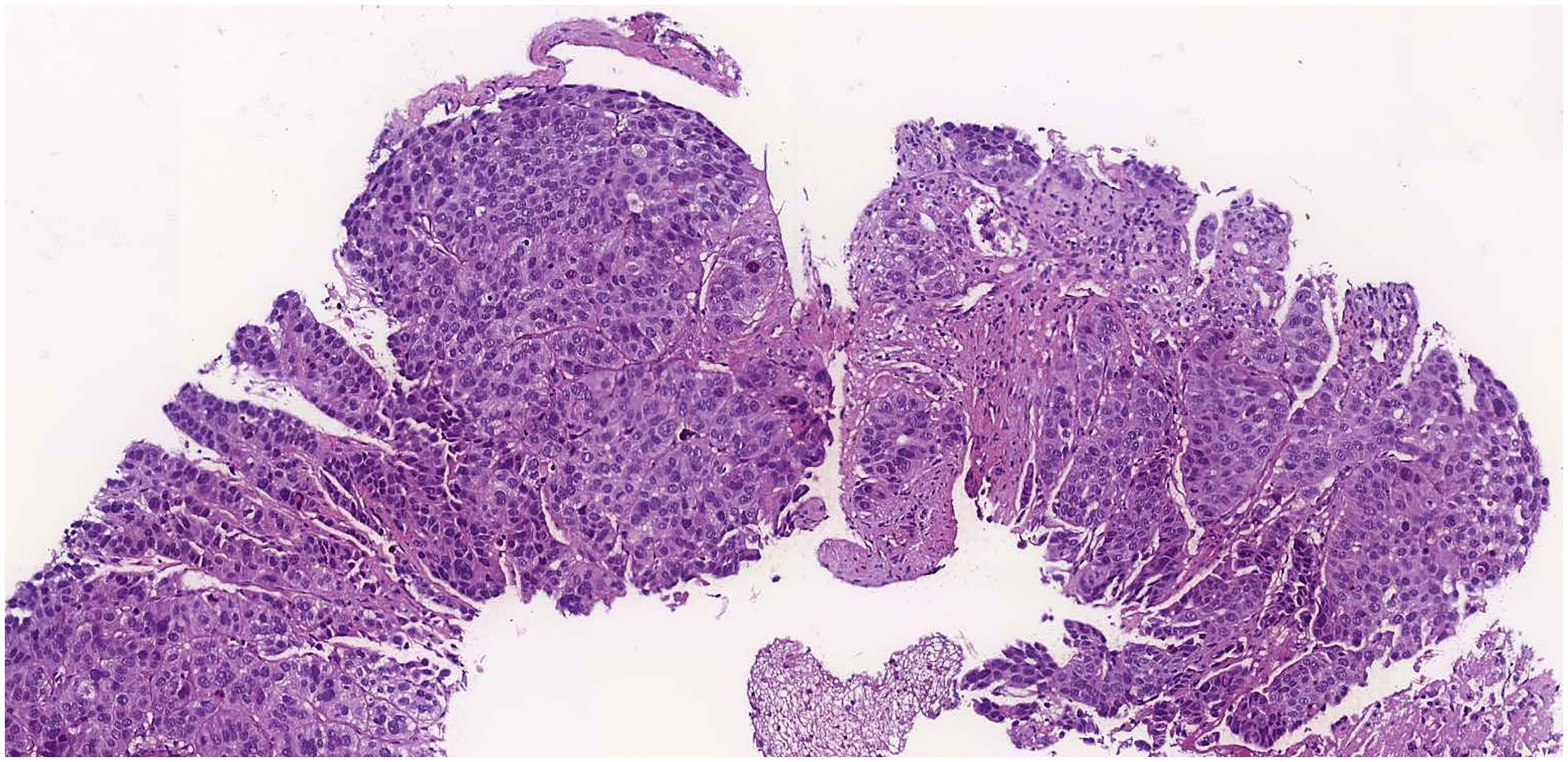
Hematoxylin eosin staining of the lesion showing carcinoma.

**Figure 2:**
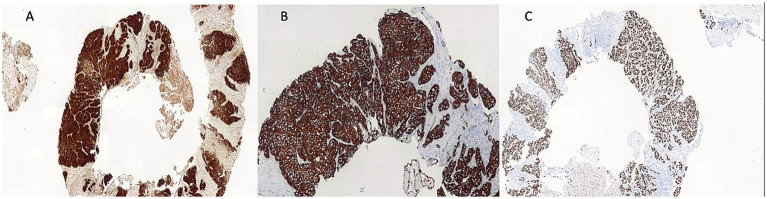
Immunoreactivity for P16 **(A)**, cytokeratin (CK) 5/6 **(B)** and P63 **(C)**.

An ultrasound of the region identified superficial venous system ectasia, marked edema of the skin and subcutaneous tissue, and a solid, hypoechoic, heterogeneous lesion with irregular contours in the right inguinal region, measuring 4.0 × 3.8 × 4.0 cm. The lesion exhibited Doppler vascularization and compressed adjacent iliac vessels, though the blood flow was preserved. A biopsy of the expansile lesion in the right hip and base of the thigh revealed an invasive, poorly differentiated carcinoma with necrotic areas (Figure 1). Immunohistochemical analysis showed positivity for p16 (Figure 2A), cytokeratin 5/6 (Figure 2B), PAX-8, and p63 (Figure 2C), consistent with invasive squamous cell carcinoma. Given this finding, we investigated the primary tumor site.

**Figure 3:**
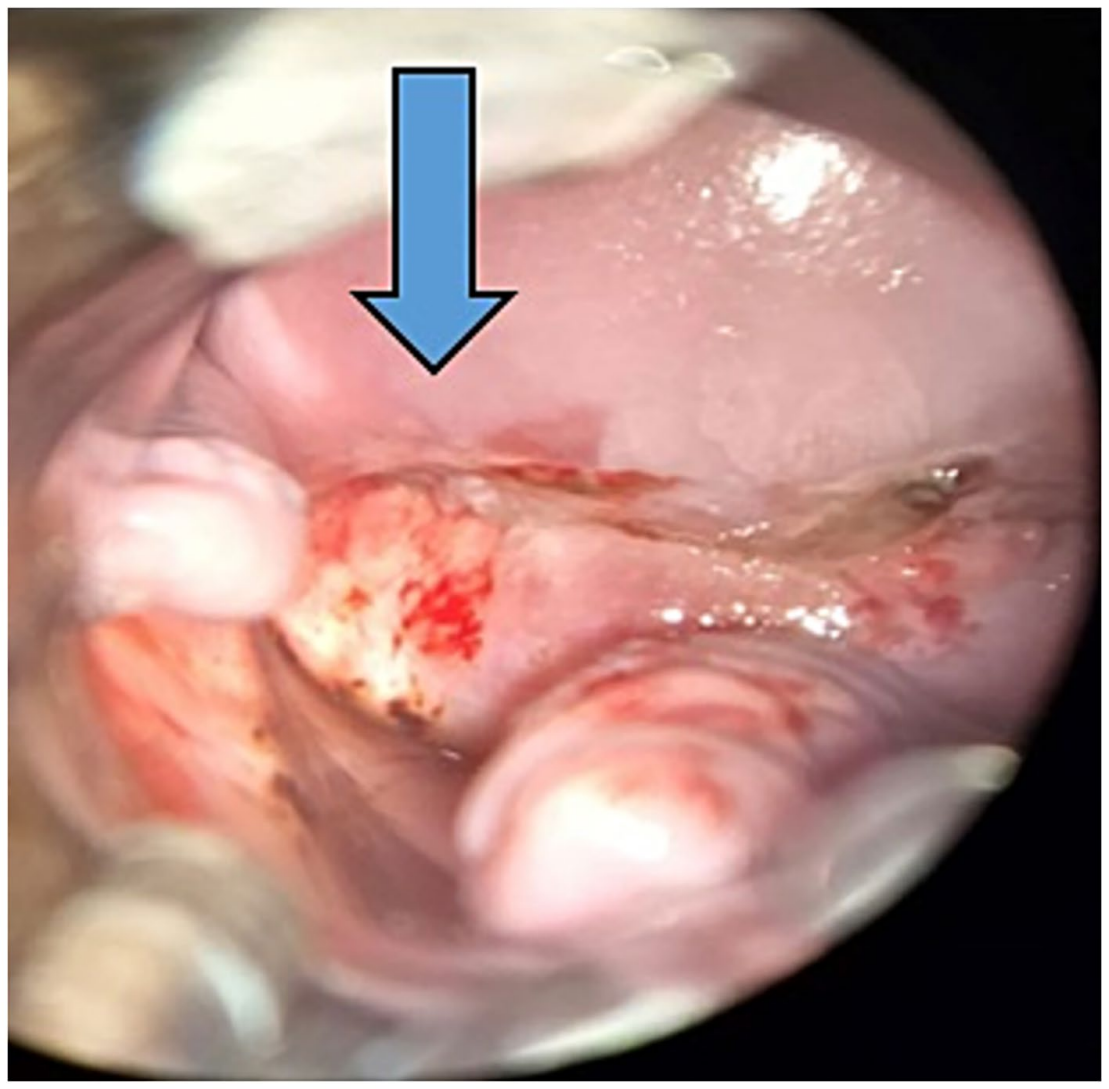
Colposcopy: cervix exhibited friable epithelium pointed by the arrow.

On physical examination, the cervix appeared normal. The patient reported a normal Pap smear 1 year before, and a new test was performed, which was again negative for malignancy. Two other potential primary sites were investigated, including the colorectal and bladder regions. Anoscopy revealed no lesions, and the urinary tract ultrasound findings were unremarkable. Due to the high suspicion of primary cervical focus, colposcopy was performed (Figure 3). No alterations were observed in the vulva or vaginal walls. The cervix exhibited friable epithelium; however, no invasive lesions were apparent. A biopsy was deferred, and a repeat Pap smear along with HPV-DNA testing were conducted.

The new Pap smear confirmed invasive squamous cell carcinoma, and HPV-DNA testing was positive for subtype 16 and other high-risk A subtypes (31, 33, 52, or 58). For staging, an abdominal and pelvic MRI was performed, revealing an exophytic nodular lesion on the right posterolateral lip of the cervix with marked diffusion restriction, measuring approximately 1.8 cm and located 2.8 cm from the internal cervical os, consistent with a primary neoplasm. The lesion involved the posterior cervical stroma. A large neoplastic lymph node conglomerate was observed in the right external iliac chain, measuring 11.0 cm in the craniocaudal direction, 8.3 cm laterally, and 5.8 cm anteroposteriorly, involving the right ureter and external and internal iliac vessels, with compression of the external iliac vein. Signs of invasion of the sacral plexus, iliac and internal obturator muscles, as well as muscle denervation were present. Additionally, the iliac bone was invaded at two points, with cortical rupture. Medially, the mass compressed the right lateral uterine wall but without evidence of invasion. Other suspicious lymph node enlargements were noted in the right common and external iliac chains, the largest measuring 4.7 × 3.0 cm. Upon confirmation of the primary tumor site, the patient was evaluated by the clinical and gynecological oncology teams.

Given the tumor’s extent, and with the informed consent of the patient, pregnancy termination was recommended to initiate immediate oncologic treatment with radiotherapy and chemotherapy. A hysterotomy was performed via a longitudinal body-fundal uterine incision of approximately 4 cm, allowing for the extraction of the fetus and placenta with intact membranes, without complications. The combined weight of the fetus, placenta, and amniotic fluid was 462 g. The patient was discharged 48 h postoperatively for oncologic treatment.

The macroscopic evaluation of the surgical specimens obtained showed normal-appearing placenta, umbilical cord, and unaltered chorionic membranes, and a male fetus, weighting 90 grams, without external or internal malformations. The histopathological examination confirmed a placenta consistent with the gestational age. The male fetus was consistent with the gestational age. Immunohistochemical analysis of the placenta showed no evidence of neoplastic involvement.

## Discussion

Cervical cancer screening using Pap smear has a false-negative rate ranging between 2 and 50% (4). In cases of negative Pap smear results despite a high clinical suspicion of cervical cancer, it is crucial that colposcopy should be considered as a complementary diagnostic tool, as demonstrated in this report. The two most common histological types of cervical cancer are squamous cell carcinoma (80%) and adenocarcinoma (7). In the present case, the patient had a normal Pap smear result, and the initial diagnosis of squamous cell carcinoma was made via biopsy of a tumor located in the inguinal region.

The primary symptoms of cervical cancer include genital bleeding, dyspareunia, abdominal pain, pelvic pain, and abnormal vaginal discharge (2). However, the patient in this study did not present with these symptoms. Instead, the patient sought medical care due to progressive edema in the right lower limb associated with severe localized pain. During the diagnostic process, a biopsy of the inguinal tumor led to the diagnosis of cervical cancer. Unilateral lower limb edema may be indicative of lymph node metastases causing lymphatic obstruction, with the most common primary tumors in women being skin cancer of the lower limbs (8), gynecological cancers (9), and anal cancer (10). The biopsy revealed squamous cell carcinoma with positive immunohistochemistry for p16, leading to the main suspicion of gynecological malignancies, such as vulvar or cervical cancers. The expression of p16 is strongly associated with HPV infection, particularly in squamous cell carcinomas, which indicates a primary HPV-related etiology, which is important for diagnosis and prognosis (11). Furthermore, the coexpression of p63 and cytokeratin 5/6 (CK5/6) is highly indicative of squamous cell carcinoma. Kaufmann et al. described that this coexpression has a sensitivity of 0.77 and specificity of 0.96 for squamous cell carcinoma (12). Two other potential primary sites were investigated, including the colorectal and bladder regions. Anoscopy revealed no lesions, and the urinary tract ultrasound findings were unremarkable.

The main suspicion in our case would be a gynecological malignancy, more specifically cervical or vulvar cancer. However, given the absence of vulvar or vaginal lesions, cervical cancer was the primary suspicion, despite the negative Pap smear result. False-negative Pap smear results can occur due to multiple factors, including the presence of adenocarcinoma, which is more challenging to detect via conventional Pap smear and depends on the quality of laboratory analysis (13). Other factors contributing to these results include improper sample collection, scant cellularity, obscuring blood and inflammation, the presence of lubricant, and errors in cytological interpretation (14). Moreover, hormonal, and vascular changes typical of pregnancy can induce reactive and inflammatory cytological alterations, which complicate access to the transformation zone (15). These factors make smear interpretation more challenging, increasing the risk of both false-positive and false-negative results.

Castle et al. demonstrated that, despite multiple screening methods—including Pap smear and HPV-DNA testing—16 out of 10,049 women diagnosed with cervical cancer were only identified during follow-up rather than during the initial screening (16). Macios et al., in a cervical cancer screening program in Poland, found that of 1,460 cases of invasive cervical cancer, 399 cases had been preceded by normal cytological results (13).

Therefore, in cases of high clinical suspicion of cervical cancer, even with a negative Pap smear result, the American Society for Colposcopy and Cervical Pathology recommends colposcopy, as it may detect lesions that Pap smear fails to identify (17). Moreover, screening combined with cervical Pap smear and high-risk-HPV detection has been used in some countries. Liu et al. studied the diagnostic value of colposcopy in cases of negative Pap smear with positive high-risk HPV results and demonstrated that colposcopy evaluation is more accurate in patients with positive high-risk HPV 16/18 or with a high viral load for quantitative HPV detection, contributing to diagnostic approach (18).

No case reports were found in the literature describing a diagnosis of cervical cancer in a pregnant or non-pregnant patient with negative Pap smear where the initial diagnosis was based on a metastasis in the inguinal tumor, lymph node in this case.

The incidence of cervical cancer during pregnancy ranges between 1.5 and 12 cases per 100,000 pregnancies (19). Since no specific guidelines have been established to manage cervical cancer during pregnancy, due to the rarity of this condition and the lack of large-scale randomized studies, the choice of the best treatment during this gestational period ends up being limited, and treatment is generally based on the protocols for non-pregnant patients, considering the clinical stage of the disease, the patient’s preferences, and fetal viability, as in this study. Treatment should be initiated promptly, as delays may lead to worse maternal outcomes.

Additionally, Tang et al. reported that when cervical cancer is appropriately managed during pregnancy, the oncological outcomes are comparable to those in non-pregnant patients (20).

Treatment of cervical cancer will depend on the cancer stage, and consists of surgery, chemoradiation, or a combination of these treatments (2). The standard treatment for patients with locally advanced cervical cancer is a combination of chemotherapy and radiation (2, 21). However, radiotherapy is contraindicated during pregnancy as it may cause fetal malformations or miscarriage. In the present case, after a multidisciplinary evaluation by the clinical and gynecologic oncology teams, the recommended treatment was radiotherapy and chemotherapy due to the extent of the tumor. If the patient wishes to continue with the pregnancy, neoadjuvant chemotherapy is offered as an option (21). However, the patient chose to terminate the pregnancy to receive the standard treatment that offered the best prognosis for her.

Following the patient’s informed consent, the pregnancy was terminated to allow for immediate initiation of oncological treatment with radiotherapy and chemotherapy.

The clinical studies suggested that in cases of invasive cervical cancer, cesarean section is preferred to vaginal delivery due to the risks of hemorrhage and to avoid the spread of cancer cells by cervical dilation or the episiotomy site, which could facilitate recurrence. Additionally, a corporeal uterine incision minimizes contact with the cervical cancer and reduce the risk of cancer cell dissemination through the uterine scar (21). Thus, cesarean section with a corporeal uterine incision was the best option and was performed in this study.

The clinical manifestations for the diagnosis of cervical cancer may vary among individuals. In our case it was performed through an inguinal tumor. This is a single case, and we did not find any other case described in the literature like ours. This limited the possibility of comparing the diagnostic and treatment management, and to draw definitive conclusions or generalizability to broader patient populations.

This case underscores the importance of colposcopy in specific cases even with negative screening test results.

## Conclusion

Colposcopy should be considered a crucial complementary diagnostic tool in patients with a high clinical suspicion of cervical cancer, even with negative Pap smear results. Additionally, prenatal care for pregnant patients with cervical cancer requires a multidisciplinary and specialized approach, ideally in a tertiary care institution, to optimize maternal and fetal outcomes.

## Data Availability

The raw data supporting the conclusions of this article will be made available by the authors, without undue reservation.

## References

[1] Guevelou J Selleret L Laas E Lecuru F Kissel M. Cervical cancer associated with pregnancy: current challenges and future strategies. Cancers. (2024) 16:1341. doi: 10.3390/cancers16071341 38611019 PMC11011172

[2] Abu-Rustum NR Yashar CM Arend R Barber E Bradley K Brooks R. NCCN guidelines® insights: cervical Cancer, version 1.2024. J Natl Compr Cancer Netw. (2023) 21:1224–33. doi: 10.6004/jnccn.2023.0062, PMID: 38081139

[3] Datir SG Jaiswal A. Cervical cancer and its association with pregnancy. Cureus. (2024) 16:e62144. doi: 10.7759/cureus.62144, PMID: 38993407 PMC11238746

[4] Diretrizes Brasileiras para o Rastreamento do Câncer do Colo do Útero. Rastreamento do Câncer do Colo do Útero (Diretriz Brasileira). Brasília: Ministério da Saúde, Instituto Nacional de Câncer José Alencar Gomes da Silva (INCA) (2022).

[5] do Nascimento Tavares SB Amaral RG Manrique EJC de Sousa NLA de Albuquerque ZBP Zeferino LC. Controle da Qualidade em Citopatologia Cervical: Revisão de Literatura. Rev Bras Cancerol. 53:355–64. doi: 10.32635/2176-9745.RBC.2007v53n3.1803

[6] Costa MCO Melo CMS Lima ES Cunha JCR Serejo APM Morais HA. Factors that cause false-negative results in oncotic cytology exams: an integrative review. Factores que causan resultados falso-negativos en exámenes de citología oncótica: una revisión integrativa. Res Soc Dev. (2021) 10:19079. doi: 10.33448/rsd-v10i10.19079

[7] Andrade LA Triglia RM. (2019). Útero – Colo uterino. Sociedade Brasileira de Patologia. 5^a^ edição. Available online at: https://www.sbp.org.br/manual-de-laudos-histopatologicos/utero-colo-uterino/ (Acessed October 20, 2024).

[8] Zaren HA Copeland EM. Inguinal node metastases. Cancer. (1978) 41:919–23. doi: 10.1002/1097-0142(197803)41:3<919::aid-cncr2820410320>3.0.co;2-a, PMID: 638977

[9] Chang Y Li G Yang Z Han G Li X Zhao Y. Inguinal nodal clinical target volume delineation based on analysis of anatomical locations of normal and metastatic lymph nodes in pelvic malignant tumors. Radiother Oncol. (2023) 183:109634. doi: 10.1016/j.radonc.2023.109634, PMID: 36963443

[10] Benson AB Venook AP Al-Hawary MM Azad N Chen YJ Ciombor KK. Anal carcinoma, version 2.2023, NCCN clinical practice guidelines in oncology. J Natl Compr Cancer Netw. (2023) 21:653–77. doi: 10.6004/jnccn.2023.0030, PMID: 37308125

[11] Gadducci A Simonetti E Cosio S Fanucchi A Dolci V Laliscia C. Positive P16 immunostaining is an independent prognostic variable for disease-free survival and overall survival in patients with squamous cell carcinoma of the vulva treated with radical surgery and inguinofemoral lymphadenectomy: an Italian single center retrospective study. Anticancer Res. (2023) 43:1643–8. doi: 10.21873/anticanres.16315, PMID: 36974801

[12] Kaufmann O Fietze E Mengs J Dietel M. Value of P63 and cytokeratin 5/6 as immunohistochemical markers for the differential diagnosis of poorly differentiated and undifferentiated carcinomas. Am J Clin Pathol. (2001) 116:823–30. doi: 10.1309/21TW-2NDG-JRK4-PFJX, PMID: 11764070

[13] Macios A Didkowska J Wojciechowska U. Risk factors of cervical cancer after a negative cytological diagnosis in polish cervical cancer screening programme. Cancer Med. (2021) 10:3449–60. doi: 10.1002/cam4.3857, PMID: 33934537 PMC8124104

[14] Zhao L Wentzensen N Zhang RR Dunn ST Gold MA Wang SS. Factors associated with reduced accuracy in Papanicolaou tests for patients with invasive cervical Cancer. Cancer Cytopathol. (2014) 122:694–701. doi: 10.1002/cncy.21443, PMID: 24888458

[15] Patton A Duncan L Bloom L Phaneuf G Zafar N. Atypical squamous cells, cannot exclude a high-grade intraepithelial lesion and its clinical significance in postmenopausal, pregnant, postpartum, and contraceptive-use patients. Cancer. (2008) 114:481–8. doi: 10.1002/cncr.23949, PMID: 18980288

[16] Castle PE Rodríguez AC Burk RD Herrero R Hildesheim A Solomon D. Neither one-time negative screening tests nor negative colposcopy provides absolute reassurance against cervical cancer. Int J Cancer. (2009) 125:1649–56. doi: 10.1002/ijc.24525, PMID: 19569231 PMC2766540

[17] Perkins RB Guido RS Castle PE Chelmow D Einstein MH Garcia F. 2019 ASCCP risk-based management consensus guidelines for abnormal cervical cancer screening tests and cancer precursors. J Low Genit Tract Dis. (2020) 24:102–31. doi: 10.1097/LGT.0000000000000525, PMID: 32243307 PMC7147428

[18] Liu Y Liao J Yi X Pan Z Pan J Sun C. Diagnostic value of colposcopy in patients with cytology-negative and hr-hpv-positive cervical lesions. Arch Gynecol Obstet. (2022) 306:1161-1169, 2022–9. doi: 10.1007/s00404-022-06415-5, PMID: 35320389

[19] Stonehocker J. Cervical cancer screening in pregnancy. Obstet Gynecol Clin N Am. (2013) 40:269–82. doi: 10.1016/j.ogc.2013.03.005, PMID: 23732031

[20] Tang X Zhang X Ding Y Zhang Y Zhang N Qiu J. A long-term retrospective analysis of management of cervical cancer during pregnancy. Int *J Gynaecol Obstet*. (2024) 165:1189–98. doi: 10.1002/ijgo.15314, PMID: 38149695

[21] Rodrigo SG Calderon J Dionisi JN Santi A Mariconde JM Rosato OD. Cervical cancer in pregnancy at various gestational ages. Int J Gynecol Cancer. 31:784–8. doi: 10.1136/ijgc-2020-002189, PMID: 33931462

